# Premedication With Midazolam Nasal Spray: An Alternative to Oral Midazolam in Children

**DOI:** 10.5812/aapm.4567

**Published:** 2012-04-01

**Authors:** Ravi K Verma, Anil Paswan, Anisa De, Surendra Gupta

**Affiliations:** 1 Department of Anesthesia, Banaras Hindu University (BHU), Varanasi, India

**Keywords:** Premedication, Midazolam, Nasal Sprays, Anesthesia

## Abstract

**Background:**

Midazolam is a water soluble benzodiazepine which is frequently administered by intravenous and oral routes in our institution. Its nasal spray has become recently available.

**Objectives:**

To compare the efficacy of midazolam administered orally and by intranasal spray, with the specific objective of assessing their efficacy in terms of acceptability to the patients, whether they achieve a satisfactory sedation score, and the overall ease of inducing general anesthesia.

**Patients and Methods:**

Sixty healthy children of ASA grade I or II, aged 2–6 years who were undergoing elective surgery of approximately 30 minutes duration, were assigned to receive midazolam premedication in a randomized controlled trial. They were divided into 2 groups of 30 patients each. Group I: 30 patients received midazolam orally (parenteral solution mixed in honey). Group II: 30 patients received a commercially available midazolam nasal spray.

**Results:**

The study shows that children better accepted the drug when administered orally than when administered intranasally, although satisfactory sedation scores at 10 and 20 minutes were better in the nasal spray group than in the oral group [i.e., 6 (20%) *vs.* 0 (0%) at 10 min and 16 (53.3%) *vs.* 13 (43.3%), respectively]. Satisfactory ease of induction scores [24 (80%) *vs.* 13 (43.3%)], recovery times [11.63 ± 4.19 minutes *vs.* 25.20 ± 9.36 minutes], and post-anesthesia recovery scores were better in the nasal spray group (group II) than in the oral group (group I).

**Conclusions:**

On the basis of our study, we conclude that nasal midazolam spray is acceptable and is a good alternative to oral midazolam as premedication in the pediatric population.

## 1. Background

It is not uncommon to see an uncooperative, frightened, crying child in the pediatric surgery preoperative holding area, particularly if the child has not been sedated or premedicated. The child is afraid of the strange hospital environment, its people, and its equipment; of being separated from the parents; and of the very word “operation,” the meaning of which is not fully comprehensible to a child.

Effective pre-anesthetic medication in children helps to allay apprehension regarding anesthesia and surgery, lessen the trauma of separation from the family, and facilitate the induction of general anesthesia and quick post-anesthetic recovery.

Midazolam is a water-soluble, chemically, midazolam HCl is 8-chloro-6-(2-fluorophenyl)-1-methyl-4H- imidazo [1,5-a] [1,4] benzodiazepine hydrochloride (1). Midazolam is frequently administered through oral and rectal routes, but bioavailability is only 40% for the oral route (2). The intramuscular route is painful and has poor acceptability (3), and the intranasal route has been in practice since 1988. Through the latter, midazolam is rapidly absorbed directly into systemic circulation, with a bioavailability of 55–83% (4, 5).

## 2. Objectives

The general objective of the study was to compare the efficacy of administering midazolam orally as a syrup versus nasally as an aerosol spray. The specific objectives were to measure:

1. acceptability of the medication;
2. acceptable levels of sedation;
3. ease of induction of general anesthesia (GA) in terms of parent-child separation, mask application and/or intravenous cannulation (IV cannulation); and
4. post-anesthesia recovery.

## 3. Patients and Methods

This study included 60 healthy male and female children aged 2–6 years who were undergoing elective surgery of approximately 30 minutes duration. Approval from the institute’s ethical committee and informed consent from the parents of the children were obtained. This was a prospective, randomized, controlled trial. The subjects were allocated to 1 of 2 groups, each containing 30 children (Group I: 30 children receiving oral midazolam in doses of 0.5 mg/kg; Group II: 30 children receiving midazolam nasal spray in doses of 0.2 mg/kg). The oral formulation was prepared by adding parenteral formulations (preservative free) to honey in a total volume of 5 mL. The intranasal preparation used is commercially available and delivers 0.5 mg per metered dose. Lower doses were used with the intranasal route because better delivery and absorption were expected with this administration route, as indicated by better bioavailability.

Children excluded from the study included those with a history of allergy to midazolam, those below the age of 2 years and above the age of 6 years, those who refused to take medication, those who were on prolonged therapy with hepatic enzyme-inducing drugs, those suffering from respiratory system dysfunction (such as rhinorrhea, bronchial asthma, nasal polyps, etc.), and those with central nervous system (CNS) dysfunction, such as epilepsy and raised intracranial tension. The following observations were made:

- Ramsay sedation score:
  1. Alert, panicky, combative, fighting without clinging;
  2. Awake, moaning, anxious, fighting but consolable; 3) Drowsy, composed, calm, minor resistance; 4) Asleep, friendly sleeping, no reaction.
- Ease of induction score:
  1. Excellent: patient unafraid, cooperative, asleep;
  2. Good: patient slightly afraid and/or crying but quieted with reassurance;
  3. Fair: patient moderately afraid, crying and not quieted with reassurance;
  4. Poor: patient crying and in need of restraint.
- Recovery score:
  1. Patient apnoeic, unable to move extremities voluntarily or on command, non-responsive and with a temperature less than 35°C or more than 37°C.
  2. Patient dyspneoic or with limited breathing, unable to move extremities voluntarily or on command but responding to painful stimuli and with a temperature range of 35 to 37°C.
  3. Patient able to breathe deeply and cough effectively, can move extremities voluntarily or on command, fully awake and with a temperature range of 35 to 37°C.

A power analysis indicated that a sample size of 28 was sufficient to detect a significant statistical difference with α = 0.05 and power 1-β = 0.9. We therefore chose 30 patients for each group. For the purpose of statistical analysis, sedation scores of 1 and 2 were considered as unsatisfactory and sedation scores of 3 and 4 as satisfactory, and ease of induction scores of 1 and 2 were considered satisfactory while ease of induction scores of 3 and 4 were considered unsatisfactory. The Chi-square test and Student’s *t*-test were used to analyze the data. Fisher’s exact test was applied for acceptability, sedation scores, ease of induction scores and post-anesthesia recovery scores, and a *P* value of < 0.05 was considered significant.

## 4. Results

All patients in both groups were identical with regard to age. There were more male patients in group II compared with group I. Patients in group I were heavier [15.96 ± 3.55 *vs*. 11.77 ± 4.42 Kg] and showed better acceptability to the drug than group II patients [24 (80%) *vs*. 16 (60%)], whereas group II patients showed better recovery times compared with group I [11.63 ± 4.19 *vs*. 25.20 ± 9.36 minute] (*Table 1*).

Sedation scores were better in group II compared with group I at 10 and 20 minutes, respectively. At 10 minutes, 6 (20%) patients in group II showed satisfactory sedation scores compared with 0 in group I, and it was statistically significant (*P* < 0.05). At 20 minutes, 18 (53.33%) patients in group II showed satisfactory sedation scores compared with 13 (43.3%) in group I, although the difference was not statistically significant (*P* > 0.05) (*Table 1*).

Satisfactory ease of induction scores were higher in group II patients compared with group I (80% *vs.* 43.3%), which was statistically significant (*P* < 0.05) (*Table 2*). Recovery scores were better in group II compared with group I. Two (6.67%) patients in group I showed recovery scores of 0 against 0 in group II, 7 (23.33%) patients in group II showed recovery scores of 1 against 3 (10%) in group I, and recovery scores of 2 were similar in both groups, 25 (83.33%) in group I versus 23 (76.67%) in group II, but the difference was not statistically significant (*P* > 0.05) (*Table 3*).

**Table 1.** Demographic Data and Recovery Times

	Group I, n = 30	Group II, n = 30	t/x^2^	*P* value
Age, y, Mean ± SD	3.83 ± 1.36	4.00 ± 1.4844	0.4625	> 0.005
Sex (M:F)	13:17	22:08	5.5543	< 0.005
Body weight, kg, Mean ± SD	15.96 ± 3.55	11.77 ± 4.42	4.1441	< 0.001
Acceptability, No. (%)	24 (80)	18 (60)	2.86	> 0.05
Recovery time, min, Mean ± SD	25.20 ± 9.36	11.63 ± 4.19	7.2462	< 0.001

**Table 2.** Ease of Induction Scores

	Excellent (1)	Good (2)	Fair (3)	Poor (4)	Satisfactory	Unsatisfactory
Group I, No. (%), n = 30	8 (26.67)	5 (16.67)	13 (43.33)	4 (13.33)	13 (43.33)	17 (56.67)
Group II, No. (%), n = 30	16 (53.33)	8 (26.67)	4 (13.33)	2 (6.67)	24 (80)	6 (20)
*P* value	< 0.05	> 0.05	< 0.01	> 0.05	< 0.01	< 0.01

**Table 3.** Post-Anesthesia Recovery Scores

Recovery Score Group	0	1	2
Group I, n = 30, No. (%)	2 (6.67)	3 (10)	25 (83.33)
Group II, n = 30, No. (%)	0 (0)	7 (23.33)	23 (76.67)
*P* value	> 0.05	> 0.05	> 0.05

**Table 4.** Sedation Scores

	10 Minutes		20 Minutes	
	Unsatisfactory, No. (%)	Satisfactory, No. (%)	Unsatisfactory, No. (%)	Satisfactory, No. (%)
Group I, n = 30	30 (100)	0	17 (56.7)	13 (43.3)
Group II, n = 30	24 (80)	6 (20)	14 (46.67)	16 (53.33)
*P* value	< 0.05		> 0.05	

## 5. Discussion

Previous surgeries with possible frightening memories may be factors in causing post-operative anxiety, and amnesia of such events is desirable. In pediatric patients, post-operative maladaptive behaviors, such as new onset enuresis, feeding difficulties, apathy, withdrawal and sleep disturbances may result from anxiety before surgery, and the incidence can be as high as 60% (6).

Numerous authors have searched for the ideal pre-anesthetic medication and also for the best medication route. The premedication must be acceptable to patients, and an atraumatic route of administration should be available, in addition to the other characteristics required of such a drug (7–9).

It has been concluded that a fine aerosol would allow greater contact with absorbing mucous membranes and that the application would be less unpleasant than drops. The bioavailability with spray has been shown to be high (83%) with virtually complete absorption (7). This high bioavailability led us to attempt this route of medication.

Some authors have studied the actual mode of delivery and application device, but only a few reported any data on acceptance. Twersky (10) used the atomizer DeVilbiss to deliver 0.2 mg/kg but did not mention acceptability. Bijorkman (7) used a spray bottle and mentioned that some patients found it slightly irritating but that all found the procedure acceptable. Other authors have mentioned temporary distress, a burning unpleasant taste, stinging, sneezing, coughing, swallowing, and crying (9). Midazolam has been given to adults by nebulizer with good acceptability (7). We used the nasal spray that is commercially available and delivers 0.5 mg per metered dose. Ljungman (11) also reported nasal discomfort in children 17.38 (45%), and it was the principal reason for dropouts in their study [8.43 (19%)]. In our study, 18 (60%) children accepted the drug in the nasal spray group as opposed to 24 (80%) in the oral group.

Administration of the drug as an aerosol is complicated by the need to keep the patient’s head still for multiple applications (12). For children weighing more than 20 kg, compliance was a significant problem because movements and struggling led to some loss of the drug. This was more noticeable as any stinging sensation was more immediately apparent. Transmucosal absorption depends upon the physical and chemical properties of the drugs. Absorption would be better if a more concentrated midazolam in a lipophilic vehicle with a neutral pH were to become available, unlike the current midazolam, which is available in a hydrophilic vehicle with an acidic pH. Secretions from nasal irritation may also alter absorption.

Griffith (12) reported sedation scores to be good in 21 (87.5%) out of 24 patients in their nasal spray group, while recovery was good in 19 (70.2%). Devis (13) reported that intranasally administered midazolam in doses of 0.2–0.3 mg/kg showed satisfactory sedation in terms of parent-child separation and satisfactory ease of induction in 70% of patients and did not prolong recovery time or hospital discharge time. Bhakta *et al.* (14) concluded that 0.2 mg/kg intranasal midazolam as nasal drops is an effective method of producing anxiolysis and sedation in pediatric patients. Kain (15) reported that 0.5 mg/kg midazolam resulted in a significant reduction in procedural anxiety in young children. In our study, sedation scores, ease of induction scores and recovery scores were more satisfactory in the nasal spray group (*Tables 2–4*). Several studies reported that orally-administered midazolam is associated with delayed discharge (16, 17). Similarly, our study found that recovery times were better in the midazolam nasal spray group than in the oral midazolam group (*Table 1*).

On the basis of the above study, midazolam nasal spray was shown to have improved sedation scores, ease of induction and recovery scores. However, its use may be limited by nasal discomfort, for which the oral route may be preferred until a more concentrated spray with lipophilic vehicle and neutral pH becomes available, which would improve its acceptability.

## References

[1] Malamed SF (2002). Sedation, a guide to patient management..

[2] Riss J, Cloyd J, Gates J, Collins S (2008). Benzodiazepines in epilepsy: pharmacology and pharmacokinetics.. Acta Neurol Scand..

[3] Karl HW, Rosenberger JL, Larach MG, Ruffle JM (1993). Transmucosal administration of midazolam for premedication of pediatric patients. Comparison of the nasal and sublingual routes.. Anesthesiology..

[4] Gudmundsdottir H, Sigurjonsdottir JF, Masson M, Fjalldal O, Stefansson E, Loftsson T (2001). Intranasal administration of midazolam in a cyclodextrin based formulation: bioavailability and clinical evaluation in humans.. Pharmazie..

[5] Knoester PD, Jonker DM, Van Der Hoeven RT, Vermeij TA, Edelbroek PM, Brekelmans GJ (2002). Pharmacokinetics and pharmacodynamics of midazolam administered as a concentrated intranasal spray. A study in healthy volunteers.. Br J Clin Pharmacol..

[6] McCann ME, Kain ZN (2001). The management of preoperative anxiety in children: an update.. Anesth Analg..

[7] Bjorkman S, Rigemar G, Idvall J (1997). Pharmacokinetics of midazolam given as an intranasal spray to adult surgical patients.. Br J Anaesth..

[8] Wermeling DP, Record KA, Kelly TH, Archer SM, Clinch T, Rudy AC (2006). Pharmacokinetics and pharmacodynamics of a new intranasal midazolam formulation in healthy volunteers.. Anesth Analg..

[9] Malinovsky JM, Populaire C, Cozian A, Lepage JY, Lejus C, Pinaud M (1995). Premedication with midazolam in children. Effect of intranasal, rectal and oral routes on plasma midazolam concentrations.. Anaesthesia..

[10] Twersky RS, Hartung J, Berger BJ, McClain J, Beaton C (1993). Midazolam enhances anterograde but not retrograde amnesia in pediatric patients.. Anesthesiology..

[11] Ljungman G, Kreuger A, Andreasson S, Gordh T, Sorensen S (2000). Midazolam nasal spray reduces procedural anxiety in children.. Pediatrics..

[12] Griffith N, Howell S, Mason DG (1998). Intranasal midazolam for premedication of children undergoing day-case anaesthesia: comparison of two delivery systems with assessment of intra-observer variability.. Br J Anaesth..

[13] Davis PJ, Tome JA, McGowan FX, Cohen IT, Latta K, Felder H (1995). Preanesthetic medication with intranasal midazolam for brief pediatric surgical procedures. Effect on recovery and hospital discharge times.. Anesthesiology..

[14] Bhakta B, Ghosh BR, Roy M, Mukherjee G (2007). Evaluation of intranasal midazolam for preanasthetic sedation in paediatric patients.. Indian J Anaesth..

[15] Kain ZN, Mayes LC, Wang SM, Caramico LA, Hofstadter MB (1998). Parental presence during induction of anesthesia versus sedative premedication: which intervention is more effective?. Anesthesiology..

[16] Viitanen H, Annila P, Viitanen M, Yli-Hankala A (1999). Midazolam premedication delays recovery from propofol-induced sevoflurane anesthesia in children 1–3 yr.. Can J Anaesth..

[17] McGraw T, Kendrick A (1998). Oral midazolam premedication and postoperative behaviour in children.. Paediatr Anaesth..

